# Cranial radiotherapy predisposes to abdominal adiposity in survivors of childhood acute lymphocytic leukemia

**DOI:** 10.1186/1748-717X-8-39

**Published:** 2013-02-21

**Authors:** Adriana Aparecida Siviero-Miachon, Angela Maria Spinola-Castro, Maria Lúcia de Martino Lee, Solange Andreoni, Bruno Geloneze, Henrique Lederman, Gil Guerra-Junior

**Affiliations:** 1 UNIFESP/EPM, Department of Pediatrics, Division of Pediatric Endocrinology, Federal University of Sao Paulo, Sao Paulo, Brazil; 2 Pediatric Oncology Institute - IOP/GRAACC, Sao Paulo, Brazil; 3 UNIFESP/EPM, Department of Preventive and Social Medicine, Division of Biostatistics, Federal University of Sao Paulo, Sao Paulo, Brazil; 4 UNICAMP, Faculty of Medical Sciences, Laboratory of Investigation on Metabolism and Diabetes - LIMED, State University of Campinas, Campinas, Brazil; 5 UNIFESP/EPM, Department of Diagnostic Imaging, Federal University of Sao Paulo, Sao Paulo, Brazil; 6 UNICAMP, Faculty of Medical Sciences, Department of Pediatrics, Division of Pediatric Endocrinology, State University of Campinas, Campinas, Brazil

**Keywords:** Precursor cell lymphoblastic leukemia-lymphoma/radiotherapy, Adiposity, Abdominal fat, Lipid metabolism disorders, Insulin resistance

## Abstract

**Background:**

Advances in treatment of acute lymphocytic leukemia increased the likelihood of developing late treatment-associated effects, such as abdominal adiposity, increasing the risk of cardiovascular disease in this population. Cranial radiotherapy is one of the factors that might be involved in this process. The aim of this study was to determine the effect of cranial radiotherapy on adiposity indexes in survivors of acute lymphocytic leukemia.

**Methods:**

A comparative cross-sectional study of 56 acute lymphocytic leukemia survivors, chronological age between 15 and 24 years, assigned into two groups according to the exposure to cranial radiotherapy (25 irradiated and 31 non-irradiated), assessed according to body fat (dual energy X-ray absorptiometry), computed tomography scan-derived abdominal adipose tissue, lipid profile, and insulin resistance.

**Results:**

Cranial radiotherapy increased body fat and abdominal adipose tissue and altered lipid panel. Yet, lipids showed no clinical relevance so far. There were significantly more obese patients among those who received cranial radiotherapy (52% irradiated versus 22.6% non-irradiated), based on dual energy X-ray absorptiometry body fat measurements. Nonetheless, no association was observed between cranial radiotherapy and body mass index, waist circumference, waist-to-height ratio or insulin resistance.

**Conclusions:**

Adolescent and young adult survivors of childhood acute lymphocytic leukemia showed an increase in body fat and an alteration of fat distribution, which were related to cranial radiotherapy. Fat compartment modifications possibly indicate a disease of adipose tissue, and cranial radiotherapy imports in this process.

## Background

Acute lymphocytic leukemia (ALL) is the most prevalent cancer in children and accounts for 80% of childhood leukemia [1]. In the past 20 years, survival rates have markedly increased with advances in treatment, and a significant number of late effects, secondary to therapy, such as weight gain, growth retardation and metabolic syndrome, have emerged [2-6].

Various mechanisms of weight gain have been reported in this group of patients, including premature adiposity rebound, growth hormone deficiency, leptin insensitivity and other factors related to lifestyle and genetics [2,4,7,8]. Nonetheless, some studies have not characterized obesity by means of body mass index (BMI) in ALL survivors, despite exposure to cranial radiotherapy (CRT) [8,9], even though an increase in BMI has often been described in this group of patients [2-4,6].

In addition to BMI, which is an established and useful marker of adiposity, a more detailed evaluation of body fat regional distribution is desired. Specifically, an excess accumulation of visceral fat within the abdomen is strongly and independently associated with cardiovascular morbidity and mortality in general population [10]. CRT has been identified as a possible factor contributing to modifications in body composition among ALL survivors [2,3,6,11,12]. To date, a more meticulous analysis of adiposity is essential because alterations in fat distribution might represent a causal link between ALL and premature cardiovascular disease, which has been previously documented in this group of patients, particularly those who received CRT [13,14].

Therefore, the purpose of this study was to assess the impact of CRT on overall adiposity and body fat distribution in young survivors of childhood ALL. Alternatively, the relationship between CRT and metabolic panel was evaluated.

## Methods

### Study population

This was a comparative cross-sectional study of a randomly selected sampling of ALL survivors of both genders from the Pediatric Oncology Institute (Federal University of Sao Paulo, Brazil), admitted from May 1991 to June 2003. The study was approved by the Ethics Research Committee of Federal University of Sao Paulo (No. 1197/07). To participate in this study, patients or parents, when appropriate, signed an informed consent form.

ALL subjects completed the Brazilian Cooperative Group for Treatment of Childhood Acute Lymphocytic Leukemia (GBTLI) international protocol. Details comprising drugs and CRT can be read elsewhere [15,16]. CRT was applied prophylactically to those at high risk for relapse, defined accordingly to the recommendations of the GBTLI, and/or therapeutically to those with central nervous system involvement, defined as blast cells greater than 5% in cerebrospinal fluid [15,16].

The inclusion criteria comprised chronological age between 15 and 24 years, the complete clinical remission of ALL (complete absence of the disease in bone marrow and blood), no ALL therapy for at least two years, complete pubertal development (menarche in girls and Tanner stage IV or above in boys) [17,18], growth of less than 1 cm/year, bone age with full epiphyseal plate fusion, normal renal, thyroid, gonadal and adrenal profiles (spontaneous or under hormonal replacement therapy), and insulin-like growth factor-1 within the normal limits. Patients who used anorexigens, insulin-sensitivity medications or other drugs that interfere with adiposity (e.g., metformin, sibutramine, and fluoxetine); who had experienced exogenous growth hormone administration within two years prior to study enrollment or a bone marrow transplantation; and who were pregnant, were postpartum, or had Down’s syndrome were excluded from the study.

Survivors were stratified into two groups according to the exposure to CRT (Yes or No). Characteristics of host/disease and therapy were assessed from clinical examinations and/or medical records, encompassing: sex, year of the GBTLI protocol employed, age at ALL diagnosis, age at assessment, age and dose of CRT (if employed), time since CRT, time post therapy, BMI Z score at ALL diagnosis, medications or eventful past medical history.

### Variables

Adiposity indexes and metabolic profile were assessed in ALL survivors at least 2 years post therapy. The BMI Z score was evaluated at different moments of therapy: diagnosis, treatment end, 2 years post treatment (period of adiposity rebound), and current.

### Adiposity indexes

#### Body composition variables

BMI and fat mass index (FMI). BMI was calculated as the weight in kilograms divided by height in meters squared (kg/m^2^) analysed in absolute values and/or converted into Z scores, based on the National Center for Health Statistics 2000 Centers for Disease Control and Prevention growth curve charts [19]. Overweight and obesity were defined as a BMI (absolute values) above 25 and 30 kg/m^2^, respectively, or a BMI Z score above 1.0 standard deviation (SD) and 2.0 SD, respectively [19,20].

The body fat mass was assessed using dual energy X-ray absorptiometry equipment (DXA), Hologic Discovery 4500 (QDR-4500A; Hologic Inc., Bedford, MA, USA), according to a method described elsewhere [21]. The FMI was calculated as the fat mass (in kilograms) divided by height in meters squared (kg/m^2^). Total body fat (in percentage) above the 95^th^ percentile was defined as obesity in patients under 18 years of age [22], and cut-offs based on the National Health and Evaluation Survey were used for patients at or above 18 years old (35.2% and 30.3% for females and males, respectively) [23].

#### Fat distribution indexes

Waist circumference (WC), hip circumference (HC), waist-to-hip ratio, waist-to-height ratio and abdominal adipose tissue, encompassing total adipose tissue (TAT) and its two layers: visceral adipose tissue (VAT) and subcutaneous adipose tissue (SAT). WC and HC were measured according to the methods described elsewhere [24], and the ratios between WC and HC and WC and height were defined as the waist-to-hip ratio and the waist-to-height ratio, respectively.

The following WC cut-offs were considered for adults: males larger than 102 cm and females larger than 88 cm (above 19 years of age) on the basis of the National Cholesterol Education Program - Adult Treatment Panel III [25]; males at or above 94 cm and females at or above 80 cm (more than 16 years of age) according to the International Diabetes Federation [26]. The cut-offs were modified for adolescents as follows: WC at or above the 90^th^ percentile (at or below 19 years of age) according to the National Cholesterol Education Program - Adult Treatment Panel III (Cook et al. study) [27] and WC greater than the 90^th^ percentile (at or below 16 years of age) according to the International Diabetes Federation [28]. Data by Freedman et al. (1999) from the Bogalusa Study [29] were used to generate the WC percentiles. A waist-to-height ratio above 0.5 indicated visceral adiposity [30].

The abdominal adipose tissue was evaluated using abdominal computed tomography (CT) scans during the morning hours. The CT scan was performed using a Philips MX 8000 Dual, and the parameters for investigation were 120 kV, 150 mA, 3.24 s exposure time, and a 5 mm width measurement with the patient in a supine position with the arms positioned beside the head. An assortment of five axial images was obtained at the L_4_-L_5_ level. The volume of the TAT was calculated (in cm^3^) with a computerized system of tridimensional analysis. The VAT was determined after the delineation of the abdominal cavity, including retroperitoneal, omental and mesenteric adipose tissue. The SAT was obtained by the difference between the TAT and VAT. The ratio between the VAT and SAT was also obtained.

### Metabolic profile

Blood samples were collected after a 12-hour overnight fast, to assess the metabolic profile by measuring lipid panel, glucose, and insulin.

#### Lipid panel

Total cholesterol, high-density lipoprotein (HDL) cholesterol, and triglycerides were determined using a colorimetric enzymatic method. Low-density lipoprotein (LDL) cholesterol was calculated by the formula described by Friedewald et al. (1972) [31]. Concerning HDL cholesterol and triglyceride levels, the following cut-offs were considered as dyslipidemia: HDL cholesterol < 1.0 mmol/L (< 40 mg/dL) and triglycerides > 2.3 mmol/L (> 200 mg/dL) [32].

#### Glucose, insulin and insulin resistance

Fasting glucose was assayed using an automated method and insulin levels in duplicate using ACTIVE® Insulin ELISA DSL-10-1600, Diagnostics Systems Laboratories, Inc., Webster, Texas, USA, having an intra- and inter-assay coefficient of variability of 2% and 5%, respectively, minimum detection limit of 1.8 pmol/L (0.3 μIU/mL), and specificity of 100%. Altered glycemia was defined according to Genuth et al. [33]. To establish insulin sensitivity, glucose and insulin levels were determined to calculate the homeostatic model assessment - insulin resistance (HOMA1-IR) [34]. With the aim of defining insulin resistance, a HOMA1-IR > 2.7 was assigned as the cut-off [35].

#### Convertion factors to système international (SI)

Total cholesterol (1 mg/dL = 0.0259 mmol/L); LDL cholesterol (1 mg/dL = 0.0259 mmol/L); HDL cholesterol (1 mg/dL = 0.0259 mmol/L); triglycerides (1 mg/dL = 0.0113 mmol/L); glucose (1 mg/dL = 0.0555 mmol/L) and insulin (1 μIU/mL = 6.945 pmol/L).

### Statistical analysis

Means and SDs were used to summarize the numerical variables and frequency counts and percentages to describe the categorical variables of the sampled subjects’ characteristics, according to CRT exposure. Fisher’s exact test was used to compare the distribution of the categorical variables between CRT levels. T-tests for independent samples were used to compare the means of continuous variables between CRT levels.

The relationship between the body composition, fat distribution and metabolic profile variables and CRT treatment was evaluated through linear regression models. Initially, regression models having body composition, fat distribution and metabolic profile as dependent variables; and exposure to CRT, sex and the interaction of CRT and sex as independent variables were evaluated. If the interaction term was significant, CRT effect was evaluated according to sex. If the interaction term was not statistically significant, it was removed from the model and the main effects of sex and CRT were tested. Similarly, if sex was not significant, it was removed from the model and CRT main effect tested. If sex was significant, the main effect of CRT adjusted for sex was reported.

A general multivariate linear model was used to compare the BMI Z score profiles along different moments of ALL therapy (diagnosis, treatment end, 2 years post therapy and current) between CRT levels. This analysis included an adjustment for age at diagnosis, which was centered at 7.5 years.

The significance level was set at 0.050. The statistical analyses were performed using SPSS 13.0 (SPSS, Inc., Chicago, IL).

## Results

### Subjects’ characteristics

The sample comprised 56 ALL survivors aged [mean (SD)] 18.6 (2.5) years who were 7.5 (3.9) years of age at ALL diagnosis and 8.5 (3.5) years post therapy. The ALL subjects completed the GBTLI international protocol, as described in Table 1. Males encompassed 42.9% of the population and 44.6% of the ALL survivors received CRT treatment with a dose of either 18 Gy (76%) or 24 Gy (24%) at a mean age of 8.0 (4.0) years. ALL subjects exposed to CRT were older at study assessment while compared to those not exposed (*T*-test, p = 0.034). Three subjects also received alternative protocols to the GBTLI, which were employed due to medullar or testicular relapses but without complementary CRT. Among the irradiated survivors, one subject also received spinal irradiation, and two patients received testicular radiotherapy (24 Gy). See Table 1 for further data.

**Table 1:** Subjects’ characteristics from 56 survivors of childhood ALL, according to CRT treatment

	**Total**				**Exposure to CRT**								**p value**
					**No**				**Yes**				
	**(n = 56)**				**(n = 31)**				**(n = 25)**				
**Variable**	**Count**		**%**		**Count**		**%**		**Count**		**%**		**Fisher’s exact test**
**Sex**													0.590
**Male**	24		42.9		12		38.7		12		48.0		
**Female**	32		57.1		19		61.3		13		52.0		
**GBTLI protocol**													0.055
**-85**	9		16.1		3		9.7		6		24.0		
**-93**	35		62.5		18		58.1		17		68.0		
**-99**	12		21.4		10		32.2		2		8.0		
**BMI Z score at ALL diagnosis**													0.862
**Normal**	46		82.1		26		83.9		20		80.0		
**Overweight**	6		10.7		3		9.7		3		12.0		
**Obese**	1		1.8		0		0.0		1		4.0		
**NA**	3		5.4		2		6.4		1		4.0		
**Variable**	**Mean**	**SD**	**Min**	**Max**	**Mean**	**SD**	**Min**	**Max**	**Mean**	**SD**	**Min**	**Max**	***T*-test**
**Age at ALL diagnosis (years)**	7.5	3.9	2.1	17.0	7.4	4.0	2.1	17.0	7.5	3.8	2.3	13.8	0.958
**Age at assessment (years)**	18.6	2.5	15.0	22.9	18.0	2.5	15.0	22.9	19.4	2.3	15.0	22.8	0.034
**Age at CRT (years)**	8.0	4.0	2.6	14.4	..	..	..	..	8.0	4.0	2.6	14.4	..
**Time post therapy (years)**	8.5	3.5	2.5	16.4	8.1	3.5	2.5	14.9	9.0	3.4	2.6	16.4	0.350
**Time since CRT (years)**	11.4	3.8	4.0	18.7	..	..	..	..	11.4	3.8	4.0	18.7	..
**BMI Z score at ALL diagnosis**	-0.28	1.38	-5.39	2.44	-0.61	1.44	-5.39	1.19	0.12	1.22	-2.30	2.44	0.055

*ALL* Acute lymphocytic leukemia, *CRT* Cranial radiotherapy, *GBTLI* Brazilian Cooperative Group for Treatment of Childhood Acute Lymphocytic Leukemia, *BMI* Body mass index, *NA* Not available, *SD* Standard deviation, *Min* Minimum, *Max* Maximum.

### Body composition

Regression models showed that a higher FMI was associated with CRT (mean difference _[yes-no]_ = 2.14, p = 0.005) (Table 2).

**Table 2:** Effect of CRT on adiposity indexes and metabolic profile in 56 survivors of childhood ALL

		**Exposure to CRT**		**Effect of CRT**			
	**Total**	**No**	**Yes**				
	**(n = 56)**	**(n = 31)**	**(n = 25)**				
**Variable**	**Mean (SD)**	**Mean (SD)**	**Mean (SD)**	**Mean difference (Yes – No)**	**SE**	**95%CI**	**p**
**BMI (kg/m**^**2**^**)**	22.7 (4.3)	21.7 (3.4)	23.8 (4.9)	2.13	1.12	[-0.12; 4.38]	0.063
**BMI (Z score)**	0.07 (1.17)	-0.09 (1.20)	0.27 (1.12)	0.35	0.31	[-0.28; 0.98]	0.266
**FMI (kg/m**^**2**^**)**	6.7 (3.1)	5.8 (2.8)	7.7 (3.3)	2.14	0.73	[0.67; 3.61]	0.005
**WC (cm)**	81.1 (12.2)	78.9 (10.2)	83.8 (14.1)	4.80	3.25	[-1.71; 11.31]	0.145
**HC (cm)**	94.1 (8.0)	92.9 (7.6)	95.5 (8.4)	2.48	2.14	[-1.81; 6.78]	0.251
**Waist-to-hip ratio**	0.86 (0.08)	0.85 (0.07)	0.87 (0.10)	0.02	0.02	[-0.02; 0.06]	0.335
**Waist-to-height ratio**	0.50 (0.07)	0.48 (0.06)	0.52 (0.09)	0.04	0.02	[0.00; 0.08]	0.065
**TAT (cm**^**3**^**)**	467.0 (279.4)	374.6 (212.0)	581.5 (313.1)	206.91	70.38	[65.81; 348.01]	0.005
**VAT (cm**^**3**^**)**	109.6 (64.1)	88.8 (52.6)	135.3 (68.7)	46.42	16.21	[13.92; 78.91]	0.006
**SAT (cm**^**3**^**)**	357.4 (231.2)	285.8 (177.6)	446.3 (261.2)	160.49	58.80	[42.60; 278.38]	0.009
**VAT/SAT**	0.40 (0.30)	0.41 (0.34)	0.37 (0.25)	-0.07	0.07	[-0.21; 0.08]	0.351
**Total cholesterol (mmol/L)**	3.9 (0.9)	3.6 (0.8)	4.3 (1.0)	0.65	0.24	[0.16; 1.13]	0.010
**LDL cholesterol (mmol/L)**	2.2 (0.7)	2.0 (0.6)	2.6 (0.7)	0.57	0.18	[0.21; 0.93]	0.003
**HDL cholesterol (mmol/L)**	1.2 (0.3)	1.2 (0.3)	1.2 (0.3)	0.02	0.08	[-0.13; 0.18]	0.754
**Triglycerides (mmol/L)**	1.0 (0.5)	1.0 (0.5)	1.1 (0.5)	0.14	0.14	[-0.14; 0.42]	0.313
**Glucose (mmol/L)**	4.7 (0.4)	4.6 (0.3)	4.8 (0.4)	0.12	0.10	[-0.08; 0.31]	0.231
**Insulin (pmol/L)**	65.7 (30.8)	61.5 (27.5)	70.8 (34.2)	9.30	8.25	[-7.25; 25.84]	0.265
**HOMA1-IR**	2.01 (1.04)	1.85 (0.92)	2.20 (1.16)	0.34	0.28	[-0.21; 0.90]	0.221

*CRT* Cranial radiotherapy, *ALL* Acute lymphocytic leukemia, *SD* Standard deviation, *SE* Standard error, *CI* Confidence interval, *BMI* Body mass index, *FMI* Fat mass index, *WC* Waist circumference, *HC* Hip circumference, *TAT* Total adipose tissue, *VAT* Visceral adipose tissue, *SAT* Subcutaneous adipose tissue, *LDL* Low-density lipoprotein, *HDL* High-density lipoprotein, *HOMA1-IR* Homeostatic model assessment - insulin resistance.

**Figure 1:**
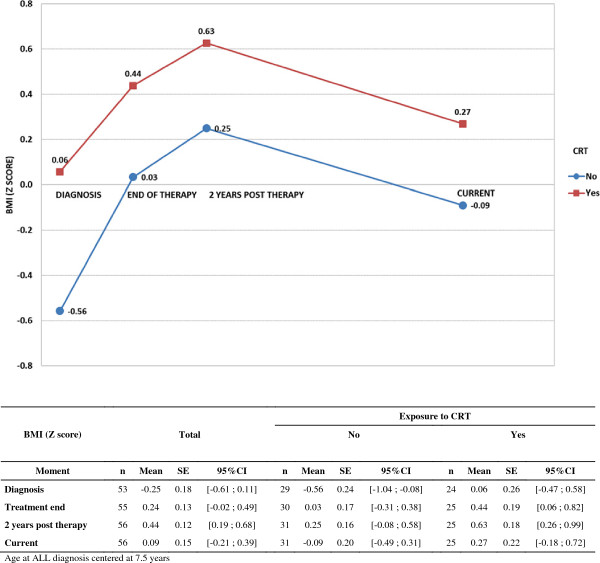
**BMI Z score along different moments of ALL therapy.** BMI Z score in 56 survivors of childhood ALL, according to the exposure to CRT, along different moments of therapy. BMI: Body mass index; ALL: Acute lymphocytic leukemia; CRT: Cranial radiotherapy; SE: Standard error; CI: Confidence interval.

At ALL diagnosis, BMI Z score was not different as regards CRT treatment (*T*-test, p = 0.055) and there was no difference in overweight or obesity concerning exposure to CRT (Fisher’s exact test, p = 0.862) (Table 1). A general multivariate linear model adjusted for age at diagnosis showed that there was a significant increase in the BMI Z score profiles along different moments of ALL therapy (diagnosis, treatment end, 2 years post therapy and current) (all mean differences p < 0.050), with no relation to the exposure to CRT (mean difference _[yes-no]_ = 0.44, p = 0.080). For further data see Table 3 and Figure 1.

**Table 3:** Comparison of BMI Z score from 56 survivors of childhood ALL along different moments of therapy

**BMI (Z score)**	**Mean Difference**	**SE**	**95%CI**	**p**
***CRT comparison***				
**Yes - No**	0.44	0.25	[-0.05; 0.93]	0.080
***Moment comparison***				
**Treatment end - Diagnosis**	0.49	0.13	[0.23; 0.74]	< 0.001
**2 years post therapy - Diagnosis**	0.69	0.15	[0.39; 0.99]	< 0.001
**Current - Diagnosis**	0.34	0.15	[0.03; 0.65]	0.032
***Age at diagnosis***	-0.10	0.03	[-0.16; -0.04]	0.001

Age at diagnosis centered at 7.5 years.

*BMI* Body mass index, *ALL* Acute lymphocytic leukemia, *CRT* Cranial radiotherapy, *SE* Standard error, *CI* Confidence interval.

On account of the total body fat measurements (by DXA), there were currently more obese patients among the survivors exposed to CRT (52% exposed to CRT versus 22.6% not exposed to CRT) (Fisher’s exact test, p = 0.020). On the basis of BMI Z score, two males were presently considered obese (3.6% of the ALL subjects and 8.3% of the male ALL subjects), and they were similarly overweight at ALL diagnosis and 2 years post therapy. There was no difference in overweight or obesity (through BMI) along different moments of ALL therapy concerning CRT treatment (Fisher’s exact test, p > 0.100).

### Fat distribution

Treatment with CRT had an effect on all of the variables derived from the CT scan: TAT (mean difference _[yes-no]_ = 206.91, p = 0.005), VAT (mean difference _[yes-no]_ = 46.42, p = 0.006) and SAT (mean difference _[yes-no]_ = 160.49, p = 0.009) (Table 2).

There was no difference between the CRT groups with respect to abdominal obesity according to the criteria of the National Cholesterol Education Program - Adult Treatment Panel III, Cook et al. or the International Diabetes Federation (Fisher’s exact test, p > 0.100). Similarly, CRT did not influence WC, HC or the waist-to-height ratio in a regression analysis (Table 2).

### Metabolic profile

Exposure to CRT increased total cholesterol (mean difference _[yes-no]_ = 0.65, p = 0.010) and LDL cholesterol (mean difference _[yes-no]_ = 0.57, p = 0.003). CRT did not influence HDL cholesterol, triglycerides or HOMA1-IR in a regression analysis (Table 2).

No patient was considered glucose intolerant or diabetic. There was no association between dyslipidemia or insulin resistance, and exposure to CRT (Fisher’s exact test, p > 0.100).

## Discussion

This study investigated whether a history of CRT, delivered as part of a complex ALL treatment, is associated with increased adiposity or adipose tissue distribution in ALL survivors. The major finding of this study is that the group exposed to CRT showed increased body fat and accelerated accumulation of fat in both layers of the abdominal region, VAT and SAT, as well as altered lipid panel. This difference in body fat and fat distribution indexes could be attributed to the disease versus therapy, including exposure to CRT, considering that survivors exposed and not exposed to CRT presented similar BMI Z score at the initiation of ALL treatment and in the first 2 years after therapy withdrawal, which is known as the critical period of the adiposity rebound.

The late effects of CRT have become an important issue in the follow-up of patients who were treated for cancer in childhood, and excessive fat accumulation has been a frequent adverse effect, especially for ALL patients during treatment, in the rebound of adiposity, and many years after therapy withdrawal [2-4,6]. However, the mechanisms by which CRT affects energy regulatory pathways and the hypothalamic metabolic circuits resulting in body fat alterations are not clear. A hypothalamic insult, such as CRT or intrathecal methotrexate, may lead to alterations in satiety centers or increase parasympathetic tone. Moreover, growth hormone deficiency, which may occur in ALL patients subjected to CRT, may have an impact on body composition and lipid levels. These alterations could lead to body fatness and metabolic derangements, both of which are factors that are suggested to contribute to features of the metabolic syndrome in this group of patients [2,4-6,11-14]. However, other factors that interact with the disease and/or therapy, such as individual host and genetic characteristics, may moderate and influence adiposity and metabolic derangements in this group of patients [3,7,8].

In the current study, FMI was selected to assess body fat and was elevated by CRT treatment, which concurs with previous data [11,12]. Abdominal CT scans were further performed to assess fat deposits directly and specifically. Prior to the Janiszewiski et al. study [11], visceral adiposity evaluations were indirect, inconsistent and limited, and they were performed using circumference measurements and DXA (trunk fat) [12,36]. Exposure to CRT increased abdominal fat in patients in this study, which concurred in previous reports [11].

There was no association between CRT and other clinically used anthropometric indexes such as BMI, WC or waist-to-hip ratio. By analysing the retrospective BMI Z score data at different moments of ALL therapy, it was evident that ALL subjects increased their BMI during treatment, particularly at the rebound of adiposity, even though it was not affected by CRT, contrary to the Childhood Cancer Survivor Study data [3], which is the largest obesity study of the United States cancer survivor population. The Childhood Cancer Survivor Study showed that the influence of CRT on the increase in BMI in ALL survivors was more evident in females exposed to CRT. To date, few patients in the present study were currently considered obese by means of BMI analysis (2 males, 8.3% of the ALL male subjects versus 13.8% from the Childhood Cancer Survivor Study at the age of 18-24 years) [3], which is in accord with other studies that have not found obesity in survivors of ALL by employing measures of BMI, despite the rebound of adiposity [8,9,37].

There may be several reasons for this lack of association between CRT and BMI in the present study: 1- a low number of subjects fulfilled the criteria for obesity based on BMI and 2- this measure is too broad and not specific enough for adipose tissue amounts. Nevertheless, BMI is a valuable clinical tool, but it does not evaluate abdominal fat deposits, which have critical clinical consequences, especially among this population, because of its implications in the risk of developing premature cardiovascular disease [12-14,38]. Additional clinical tools have been used to assess fat distribution, including circumferences and their relations. These indexes are used in epidemiological studies, as well as in the clinical setting, but they also have several limitations that make them inferior to the direct measurement of adipose tissue mass [28,30].

Concerning the lipid panel for the evaluation of cardiovascular disease risk factors, the results of this study showed increased levels of LDL cholesterol in the CRT group, to date with no clinical relevance, which is in accordance with other reports [11,12,36]. Nonetheless, HDL cholesterol, triglyceride concentrations and insulin resistance were not altered, and no influence of CRT was observed, even though these metabolic derangements had been described by various previous studies [11,12,14,36]. Alterations in body fat and metabolic panel are clearly multifactorial, being regulated by gender, host characteristics, therapeutic agents (chemotherapy and exposure to CRT) and hormonal deficiencies. Although it was not within the aim of this study, factors other than CRT could have influenced body fatness and metabolic profile in this population, such as the young age, shorter post treatment interval, overprotection due to illness, intense parental care giving, and medical support [5,6,8].

This study population included 56 survivors of ALL from a single institution, and none of the ALL subjects currently assessed was within the period of the adiposity rebound or presented any other condition that would modify body fat or the metabolic state. In addition, significant differences in BMI Z scores were not present before ALL diagnosis or treatment. Patients subjected to CRT were older than those not exposed as they may have underwent preceding GBTLI protocols (GBTLI-85 and -93) [15,16], which indicated prophylactic CRT to all patients at risk for relapse; however, this difference regarding age was not clinically relevant. Concerning CRT and its detrimental effects, the latest GBTLI-99 protocol has reserved therapeutic CRT to patients with central nervous system involvement so that patients treated for ALL are not exposed to prophylactic CRT [16]. The effects of drugs (e.g., intrathecal methotrexate) and ALL itself on the central nervous system and the implications for fatness are issues that require further study [2,39].

## Conclusions

Overall, CRT contributed to an increase in body fatness and modification of fat distribution and lipid panel in adolescent and young adult survivors of childhood ALL at a mean chronological age of 18.6 years and 8.5 years post treatment. Survivors of ALL should be followed during their lifetime to prevent the risk factors that are associated with metabolic syndrome and cardiovascular disease, such as obesity, particularly abdominal obesity. The present study also showed that simple variables such as weight and BMI are not sufficient for such a follow-up. The supplementary indexes, such as fat distribution variables, may better reflect the risk of cardiovascular disease in this group of patients and must be added. The alterations in adipose tissue deposition should be assessed by further studies to determine its true role and pathogenic potential and whether it represents a disease or a dysfunction in this group of patients.

## Abbreviations

ALL: Acute lymphocytic leukemia; BMI: Body mass index; CRT: Cranial radiotherapy; CT: Computed tomography; DXA: Dual energy X-ray absorptiometry; FMI: Fat mass index; GBTLI: Brazilian Cooperative Group for Treatment of Childhood Acute Lymphocytic Leukemia; HC: Hip circumference; HDL: High-density lipoprotein; HOMA1-IR: Homeostatic model assessment - insulin resistance; LDL: Low-density lipoprotein; SAT: Subcutaneous adipose tissue; SD: Standard deviation; TAT: Total adipose tissue; VAT: Visceral adipose tissue; WC: Waist circumference

## Competing interests

The authors declare that they have no competing interests.

## Authors’ contributions

AAS-M participated in study design, data collection and organization, as well as preparation of the manuscript; AMS-C also participated in study design, preparation and revision of the manuscript; MLML participated in data collection and organization, as well as in revision of the manuscript; SA performed the statistical analyses and revised the manuscript; BG oversaw the study, its design and conception and revised the manuscript; HL performed all evaluations of CT and revised the manuscript; GG-J oversaw the study, its design and conception and revised the manuscript. All authors read and approved the final manuscript.
